# *Porphyromonas gingivalis* outer membrane vesicles induce an activated but permissive neutrophil phenotype

**DOI:** 10.3389/fimmu.2026.1896511

**Published:** 2026-09-03

**Authors:** Ailén Fretes, Brenda Lara, Guillermina Calo, Ignacio Rojas Campión, Lourdes Materazzi, Florencia Sabbione, Stefania A. Robaldi, Maria Constanza Pautasso, Claudia Pérez Leirós, Daiana Vota, Paula M. Tribelli, Vanesa Hauk

**Affiliations:** 1 Universidad de Buenos Aires – CONICET, Instituto de Química Biológica de la Facultad de Ciencias Exactas y Naturales (IQUIBICEN)., Buenos Aires, Argentina; 2 Instituto de medicina experimental (IMEX)‐Consejo Nacional de Investigaciones Científicas y Técnicas (National Scientific and Technical Research Council)/Academia Nacional de Medicina de Buenos Aires, Buenos Aires, Argentina

**Keywords:** immune evasion, neutrophils, *P. gingivalis* outer membrane vesicles, periodontal disease, pregnancy complications

## Abstract

**Introduction:**

*Porphyromonas gingivalis* (Pg) is a keystone periodontal pathogen linked to adverse pregnancy outcomes that releases outer membrane vesicles (OMV) capable of modulating innate immune responses; however, whether PgOMV reprogram neutrophil antimicrobial and metabolic functions, and how the bacterial oxidative environment shapes these effects, remained unknown.

**Methods:**

To address this, we evaluated the impact of PgOMV on human peripheral neutrophil activation, NET formation, metabolic parameters, and intracellular bacterial internalization and persistence, as well as their indirect effects on trophoblast migration. We further compared responses to OMV derived from bacteria grown under basal versus oxidative stress conditions (H_2_O_2_-PgOMV).

**Results:**

PgOMV induced neutrophil activation, as evidenced by increased ROS production and CD11b surface expression. Despite this activation, PgOMV did not trigger NET formation and significantly reduced extracellular DNA through DNase activity, as confirmed using PMA-induced NET preparations. PgOMV stimulation increased glucose uptake and lipid droplet accumulation, reduced lactate release, and decreased both mitochondrial mass and membrane potential. Neutrophils preconditioned with PgOMV displayed significantly greater Pg intracellular internalization and persistence relative to untreated controls, and conditioned media from PgOMV-stimulated neutrophils impaired HTR-8/SVneo trophoblast migration. Comparison of basal and H_2_O_2_-PgOMV revealed that both preparations similarly activated neutrophils, but H_2_O_2_-PgOMV did not alter glucose uptake or lactate release, and neutrophils preconditioned with H_2_O_2_-PgOMV showed significantly greater *P. gingivalis* internalization and persistence beyond that seen with basal PgOMV.

**Conclusions:**

Together, these findings indicate that PgOMV induce an activated but permissive neutrophil phenotype characterized by activation, impaired extracellular antimicrobial responses, altered metabolic-associated parameters, and increased susceptibility to intracellular bacterial survival and that oxidative stress further enhances bacterial persistence. The impairment of trophoblast migration by PgOMV-conditioned neutrophil media supports an indirect, neutrophil-mediated mechanism through which periodontal-derived vesicles may contribute to placental dysfunction, with implications for the link between periodontitis and adverse pregnancy outcomes.

## Introduction

*Porphyromonas gingivalis* (Pg) is a keystone pathogen in periodontitis, a chronic inflammatory disease associated with systemic conditions including adverse pregnancy outcomes such as preeclampsia and fetal growth restriction (1). Like other gram-negative bacteria, Pg releases outer membrane vesicles (OMV), nanosized bilayered structures enriched in lipopolysaccharide, outer membrane proteins, enzymes, toxins, and nucleic acids (2, 3). We have previously shown that PgOMV regulate trophoblast inflammatory and metabolic responses (4–6) and that oxidative stress reshapes their protein cargo, enriching proteins associated with adhesion, stress adaptation and virulence and physicochemical properties (7). Emerging evidence indicates that OMV actively modulate innate immune cell metabolism and effector fate (8), yet how inflammatory environmental conditions influence these interactions remains poorly understood.

Neutrophils are the predominant immune cells recruited to the periodontium during infection, where they represent the first line of defense against Pg (9). However, Pg has developed several capacities to subvert this defense to its own advantage and to persist in the presence of neutrophils (9, 10). Notably, neutrophils from patients with chronic periodontitis display an altered activation profile, suggesting that the periodontal environment shapes their functional state (9). Yet whether PgOMV can reprogram neutrophil antimicrobial and metabolic responses has not been investigated.

Beyond the periodontium, Pg can disseminate systemically and their DNA and antigens have been detected in placental tissues and amniotic fluid from complicated pregnancies (11, 12). At the maternal-fetal interface, neutrophils also play key roles, and their dysregulation, including excessive ROS and aberrant NET formation, has been implicated in preeclampsia and placental insufficiency (13). Circulating neutrophils undergo progressive functional and metabolic changes throughout pregnancy that correlate with gingival inflammatory status, and components of gingival crevicular fluid enriched in Pg can directly activate neutrophils *ex vivo* (14). Whether OMV-conditioned neutrophils can in turn impair trophoblast behavior remains unexplored and may represent an indirect mechanism linking periodontal infection to placental dysfunction.

In this study, we evaluated the effects of PgOMV on human neutrophil activation, NET formation, metabolic parameters, and bacterial internalization and persistence, and assessed their downstream impact on trophoblast migration. We further compared neutrophil responses to OMV derived from bacteria cultured under basal versus oxidative stress conditions. Our results indicate that PgOMV induce neutrophil activation and alter neutrophil metabolic parameters while impairing antimicrobial responses, and that oxidative stress reshapes these properties in a manner that enhances intracellular bacterial internalization and persistence. PgOMV conditioned neutrophils, in turn, impaired trophoblast migration, suggesting a neutrophil-mediated indirect mechanism linking periodontal infection to adverse pregnancy outcomes.

## Materials and methods

### *Porphyromonas gingivalis* outer membrane vesicle isolation

*Porphyromonas gingivalis* ATCC 33277 was grown anaerobically at 37 °C in ATCC Medium 2722 (Tryptic Soy Broth 30.0 g/L and Yeast Extract 5.0 g/L) supplemented with 5% L‐cysteine, 5 mg/L hemin, and 1 mg/L vitamin K1. Bacteria were cultured in anaerobic conditions for a total of 5 days until it reached the early stationary phase. For the final 24 h, cultures were divided into two experimental conditions: control cultures, which continued under basal conditions, and oxidative stress–treated cultures, which were exposed to 30 mM hydrogen peroxide (H_2_O_2_),.

Cultures were harvested at early stationary phase (OD_600_: 1.0-1.2), and bacterial cells were removed by two centrifugations at 6000 × g for 15 min at 4 °C. The resulting supernatants, containing extracellular vesicles, were passed through a 0.22 µm membrane filter (GE Healthcare Life Sciences, Boston, MA, USA) to eliminate residual cells and debris. OMVs were pelleted by ultracentrifugation at 100,000 × g for 70 min at 4 °C. The resulting vesicle containing pellet was gently resuspended in PBS and purified by a second ultracentrifugation step at 100,000 × g for 70 min at 4 °C. The final pellet was re-suspended in PBS and stored at −80 °C until use.

The physicochemical characterization of both OMV preparations, including size distribution by nanoparticle tracking analysis, protein content, and morphology by transmission electron microscopy, was previously reported (7). Briefly, both preparations yielded vesicles of comparable size and morphology, confirming successful isolation.

### Human neutrophil isolation

Neutrophils from peripheral blood of healthy volunteers were obtained as approved by the Argentine Society of Clinical Investigation Ethics Committee Board (#10469/23). As detailed previously (5, 14), peripheral blood neutrophils were isolated by centrifugation on Ficoll-Paque PLUS (GE Healthcare, Uppsala, Sweden) and Dextran (MP Biomedicals, CA, USA) sedimentation. After hypotonic lysis, neutrophils were suspended in RPMI-1640 medium (Thermo Fisher Scientific) supplemented with 10% heat-inactivated fetal bovine serum (FBS, Internegocios). The purity was routinely checked by flow cytometry (>98%). Representative purity plots are shown in **Supplementary Figure 1**. Cells were used immediately after isolation.

### ROS production

For ROS production, 2x10^5^ neutrophils were stimulated 45 min with 5 μg/mL PgOMV and 5 μM 2′,7′-Dichlorofluorescin diacetate (DCFH-DA) (Sigma-Aldrich) was added for another 15 min, then cells were thoroughly washed and assessed using a BD Accuri C6 Plus cytometer and analyzed with FlowJo 10 software as before (5, 14).

### NET formation

To assess NET formation, we quantified extracellular DNA and neutrophil elastase activity. Briefly, 1x10^6^ neutrophils were first incubated in phenol red-free medium and stimulated for 210 min with 5 μg/mL PgOMV. After stimulation, cultures were exposed to DNase I (1 U/ml) for 30 min to release NET-associated components into the supernatant. Extracellular DNA was measured fluorometrically using Sytox Green (5 µM, Sigma-Aldrich), and elastase activity was determined with a 1 mM chromogenic substrate specific for human neutrophil elastase (Sigma-Aldrich) (14). As a positive control for NET formation, neutrophils were stimulated with 50 nM PMA for 210 min, which consistently induced robust extracellular DNA release and elastase activity under our experimental conditions, confirming the validity of the assay (**Supplementary Figure 2**).

To evaluate the effect of PgOMV on NETs, 1x10^6^ neutrophils were stimulated with 50 nM PMA for 210 min. Then, DNase I (1 U/ml) was added for 30 min to release NET-associated components into the supernatant. The supernatants were incubated with 5 μg/mL PgOMV for 120 min more and extracellular DNA was measured as described above.

### Lipid droplets quantification

Neutrophils were stimulated with 5 μg/mL PgOMV for 180 min, washed twice with PBS and incubated with 2 μM BODIPY 493/503 (Thermo Fisher Scientific) for 15 min at 37 °C. They were then washed with cold PBS and resuspended in 2% FBS in PBS solution for immediate flow cytometry analysis (15).

### Fluorescent metabolic dyes assays

Mitochondrial mass and membrane potential was measured in 2x10^5^ neutrophils stimulated during 180 min by using MitoSpy Green (Biolegend Inc, California, EEUU) and Mitotracker Red CMXRos (Termo Fisher Scientific, Massachusetts, EEUU), respectively. The data was acquired in a BD Accuri cytometer and was analyzed using the FlowJo software.

### Glucose uptake

Glucose uptake was assessed using the fluorescent glucose analog 2-NBDG (Thermo Fisher Scientific). Neutrophils were stimulated with 5 µg/mL PgOMV for 60 min, then incubated with 2-NBDG for 5 min at 37 °C. Cells were washed with cold PBS and analyzed by flow cytometry (15).

### Lactate release

To evaluate lactate release, 7,5x10^6^ neutrophils were stimulated for 6 h in presence or absence of 5 μg/mL PgOMV. Then, lactate concentration in cell supernatant was measured using the Lactate kit REF 1999795 (Wiener Lab, Rosario, Argentina) according to the manufacturer’s instructions.

### Long-chain fatty acid uptake

Neutrophils were cultured with BODIPY-FL C12 (Thermo Fisher Scientific), following a protocol adapted from (15). BODIPY-FL C12 is a fluorescently labeled saturated fatty acid analog containing a 12-carbon chain that mimics the behavior of an 18-carbon fatty acid. Prior to cell incubation, the probe was complexed with 0.1% fatty acid-free bovine serum albumin (Sigma-Aldrich) for 30 min at 37 °C. Cells were then washed with PBS and exposed to 5 μM BODIPY-FL C12 diluted in serum-free RPMI-1640 medium for 10 min. After incubation, cells were washed with PBS containing 0.2% BSA, resuspended in PBS supplemented with 2% FBS, and fluorescence acquisition and analysis were performed by flow cytometry.

### Wound healing assay

To obtain conditioned media, 1x10^6^ neutrophils were treated in the presence of PgOMV or absence (-) during 60 min. Then, neutrophils were washed with PBS and incubated in RPMI 1640 supplemented with 2% SFB for 5 h more and neutrophil conditioned media (CM) was collected. Human trophoblast cells HTR‐8/SVneo cell line (HTR‐8) were plated on 48‐well polystyrene plates. At confluence, a thin scratch was made in the monolayer with a sterile tip (wound) and cells were subsequently incubated for 18 h in the different settings. Microphotographs were taken at 0 and 18 h postscratching. The images were analyzed using ImageJ^®^ software and the wound closure was calculated as described (16).

### Bacterial internalization and persistence assay

Since, in professional phagocytes such as neutrophils, the gentamicin protection assay does not discriminate between bacteria internalized through phagocytosis or pathogen-driven invasion mechanisms, we refer to this outcome as bacterial internalization assay rather than invasion assay.

The internalization assay was performed as described previously (7, 17, 18). Briefly, 1x10^6^ neutrophils were treated in the presence of 5 µg/mL PgOMV or H_2_O_2_ - PgOMV or absence (-) during 2 h. Then, cells were washed with phosphate-buffered saline (PBS) without any supplements prior to incubation with *P. gingivalis*. Bacterial cells were cultured anaerobically for 3 days as described above. Bacterial suspension was adjusted to achieve a multiplicity of infection (MOI) of 30 and afterwards was incubated with host cells for 1.5 h for internalization or 24 h for persistence assays. Afterwards, to eliminate non-invasive Pg cells were treated with 300 μg/mL gentamicin (Invitrogen) for an additional 1.5 h. As a control for the gentamicin protection assay, *P. gingivalis* incubated with 300 μg/mL gentamicin for 1.5 h in the absence of neutrophils yielded no recoverable colonies, confirming the effectiveness of the antibiotic treatment in eliminating extracellular viable bacteria. For persistence assays, infected neutrophils were then incubated for an additional 24 h following gentamicin treatment. The host cells were washed twice with PBS and lysed with 0.1% Triton X-100, 0.5% trypsin, and 0.3 m g/mL DNase in PBS. Aliquots (5 μL) of several dilutions were dropped on blood agar plates (ATCC Medium 2722; Tryptic Soy Broth 30.0 g/L, Yeast Extract 5.0 g/L, and agar 15.0 g/L) supplemented with 5% L-cysteine, 5 mg/L hemin, 1 mg/L vitamin K1 and 5% ovine blood and incubated 5–7 days under anaerobic conditions at 37 °C. The number of internalized bacteria was determined based on the calculation of bacterial colonies grown on the agar plates. Bacterial persistence was calculated as the ratio of the number of bacteria recovered after 24 h to the number of internalized bacteria.

## Results

### PgOMV activate human neutrophils and increase ROS production

**Figure 1:**
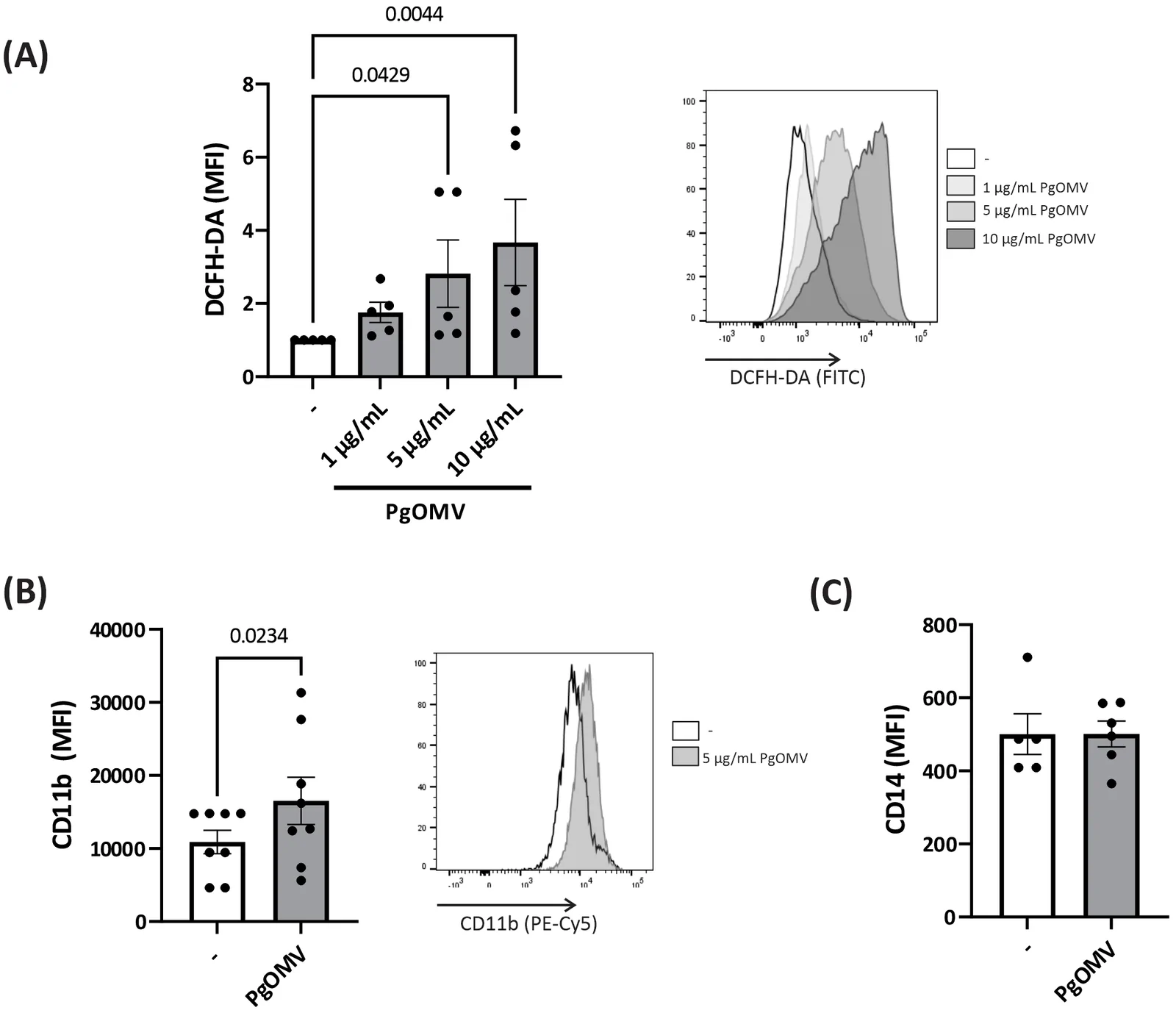
PgOMV induce activation and ROS production in human neutrophils. **(A)** Neutrophils were stimulated during 45 min with increasing concentration of PgOMV and ROS production was analyzed by flow cytometry using 2,7-dichlorofluorescin diacetate. Neutrophils were stimulated for 60 min with 5 μg/mL PgOMV and CD11b **(B)** and CD14 **(C)** expression were assessed via flow cytometry. For ROS measurements, each dot represents one independent donor (n = 5). For CD11b and CD14 analyses, neutrophils from each donor were stimulated with two independent PgOMV preparations obtained from different purification batches; therefore, two data points are shown for each donor. Biological replicates were n = 4 for CD11b and n = 3 for CD14. Values are mean ± SEM. Friedman with Dunn’s correction test was used in A, whereas Wilcoxon matched-pairs signed-rank test was used in B and C.

To determine an effective concentration for subsequent experiments, we first performed a dose-response analysis of PgOMV induced ROS production in human neutrophils across concentrations ranging from 1 to 10 µg/mL. PgOMV stimulation increased intracellular ROS in a dose-dependent manner, as measured by DCFH-DA fluorescence. The concentration of 5 µg/mL was selected for all subsequent experiments, as it represented the lowest concentration of PgOMV that produced a statistically significant increase in ROS compared to unstimulated controls (**Figure 1A**).

We next evaluated whether PgOMV modulate additional activation parameters. Surface expression of CD11b, an integrin associated with neutrophil activation and adhesion, was significantly increased in neutrophils stimulated with PgOMV compared to unstimulated controls (**Figure 1B**). We also evaluated CD14 surface expression, given that PgOMV have been reported to reduce CD14 in macrophages. In contrast to macrophages, PgOMV stimulation did not alter surface CD14 expression in neutrophils (**Figure 1C**), suggesting that these cells may sense PgOMV through different surface receptors.

### PgOMV do not induce NETosis and reduce extracellular DNA through DNase activity

**Figure 2:**
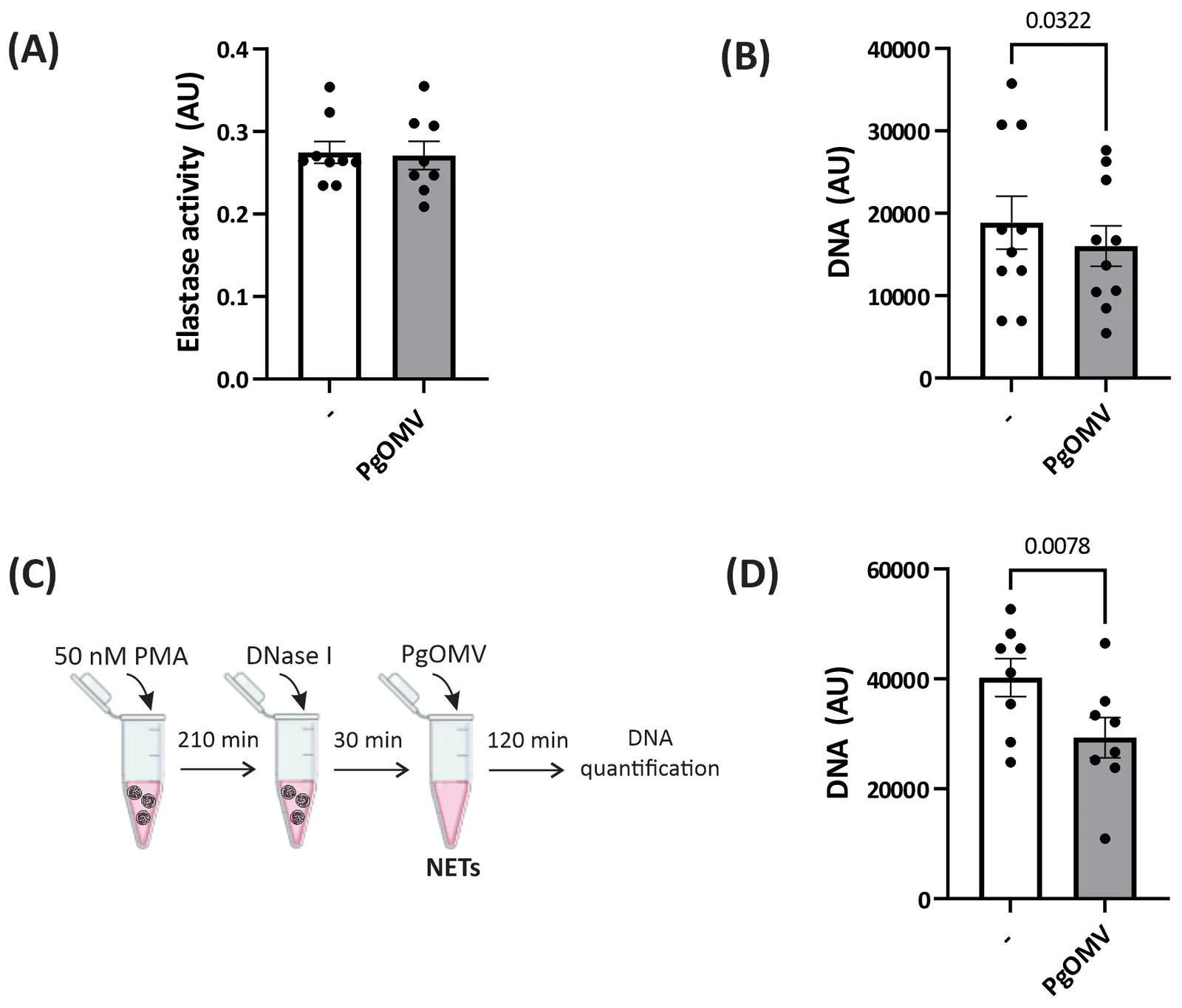
PgOMV do not induce NETosis. Neutrophils were stimulated with 5 μg/mL PgOMV during 210 min and supernatants were collected to evaluate **(A)** elastase activity and **(B)** DNA release. **(C)** To study the DNase activity of PgOMV, neutrophils were challenged with 50 nM PMA for 210 min and then **(D)** NETs-enriched supernatants were exposed to PgOMV for 120 min and DNA remanent in supernatants after PgOMV treatment was measured. For elastase and DNA release studies, neutrophils from each donor were stimulated with two independent PgOMV preparations obtained from different purification batches; therefore, two data points are shown for each donor. Biological replicates were n = 6 for elastase and n=7 for DNA release. Wilcoxon matched-pairs signed-rank test was used.

We next assessed whether PgOMV stimulation triggered NET formation by quantifying extracellular DNA by Sytox Green fluorometry and elastase activity in cell supernatants. PgOMV did not induce NETosis in human peripheral neutrophils, as evidenced by extracellular elastase levels comparable to unstimulated controls (**Figure 2A**). Strikingly, extracellular DNA was significantly reduced compared to unstimulated controls (**Figure 2B**).

The reduction in extracellular DNA prompted us to evaluate whether PgOMV have DNase activity. To address this, we induced NET formation with PMA, isolated NET enriched supernatants, and incubated them with PgOMV for 120 min (**Figure 2C**). PgOMV treatment significantly reduced extracellular DNA in these preparations (**Figure 2D**), consistent with DNase activity associated with PgOMV.

### PgOMV alter glucose handling, lipid droplet accumulation and mitochondrial parameters in neutrophils

**Figure 3:**
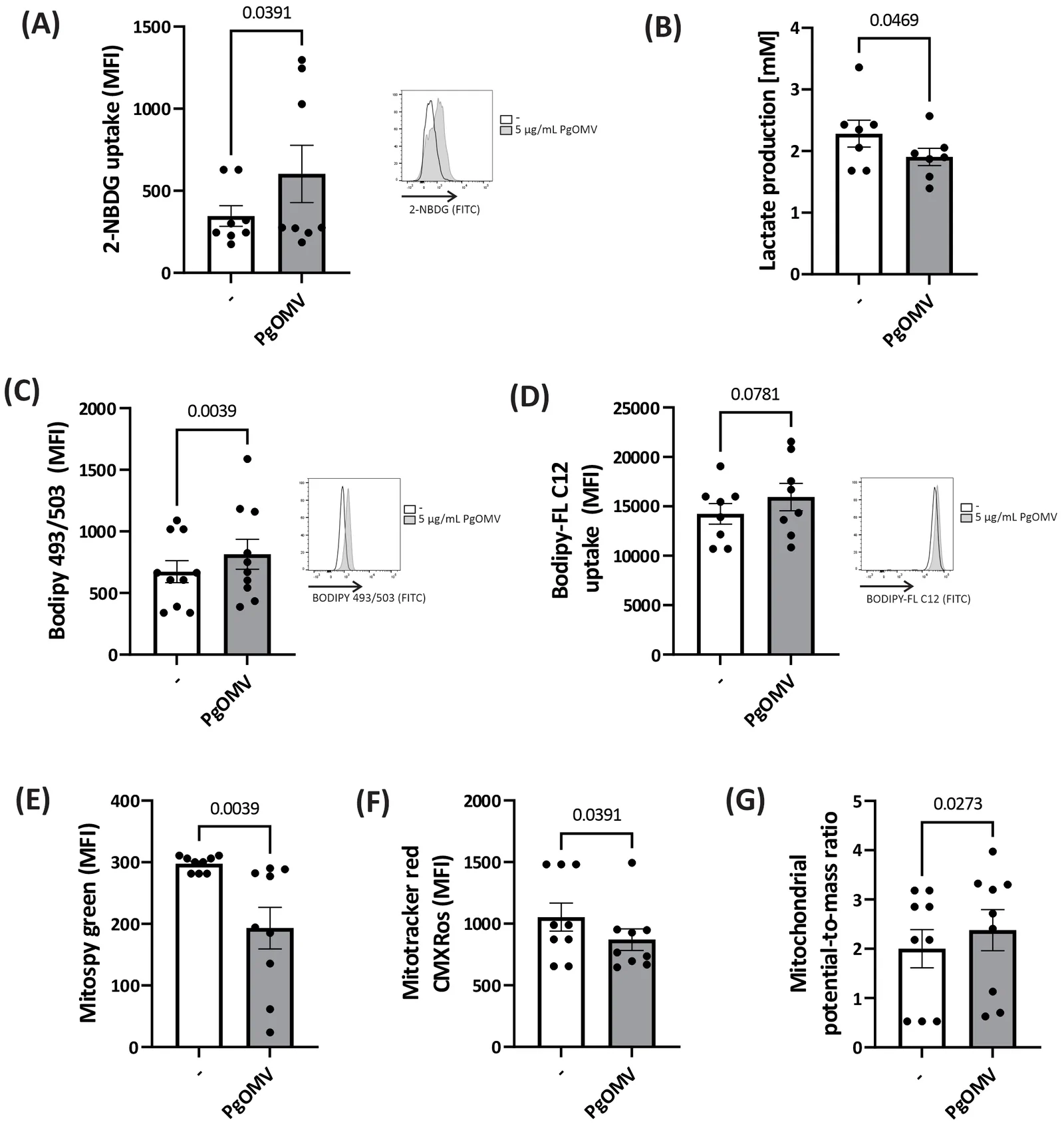
PgOMV modulate metabolic associated parameters in neutrophils. **(A)** To evaluate glucose uptake, neutrophils were stimulated with PgOMV for 60 min and then 2-NBDG uptake was evaluated. **(B)** Neutrophils were stimulated with PgOMV during 6 h and supernatant was collected to evaluate lactate production. Values are mean ± SEM. Each point represents an individual sample. (n=7 for the lactate assay) Wilcoxon matched-pairs signed-rank test was used. **(C)** Neutrophils were stimulated with PgOMV for 180 min or 60 min to evaluate lipid droplet accumulation and **(D)** long-chain fatty acid uptake by flow cytometry using BODIPY 493/503 and BODIPY-FLC12 probes, respectively. To evaluate mitochondrial mass **(E)** and mitochondrial membrane potential **(F)**, neutrophils were stained with Mitospy green and Mitotracker red CMXRos respectively and evaluated by flow cytometry. **(G)** Mitochondrial membrane potential-to-mass ratio. Results are expressed as mean ± SEM. For 2-NBDG, Bodipy 493/503, Bodipy-FL C12 uptake, mitochondrial mass, mitochondrial membrane potential, and mitochondrial potential-to-mass ratio, neutrophils from some donor were stimulated with two independent PgOMV preparations obtained from different purification batches; therefore, two data points are shown for some donor. Biological replicates were n = 6 for 2-NBDG, n = 6 for Bodipy 493/503, n = 6 for Bodipy-FL C12, and n = 5 for mitochondrial parameters. Wilcoxon matched-pairs signed-rank test was used.

We next evaluated whether PgOMV stimulation was associated with alterations in metabolic parameters related to glucose handling. PgOMV stimulation increased glucose uptake, assessed by 2-NBDG incorporation (**Figure 3A**). However, lactate release was significantly reduced in OMV-stimulated neutrophils compared to unstimulated controls (**Figure 3B**), suggesting that the increased glucose uptake was not paralleled by increased extracellular lactate accumulation under the experimental conditions evaluated. Regarding lipid metabolism-related parameters, PgOMV stimulation also increased lipid droplet accumulation as measured by BODIPY 493/503 fluorescence (**Figure 3C**) and tended to increase long-chain fatty acid uptake, as assessed by BODIPY-FL C12 incorporation (**Figure 3D**). With respect to mitochondrial parameters, PgOMV stimulation significantly reduced mitochondrial mass and membrane potential as measured by MitoSpy Green fluorescence and MitoTracker CMXRos respectively (**Figures 3E, F**). Interestingly, the mitochondrial membrane potential-to-mass ratio was increased in PgOMV-stimulated neutrophils compared to unstimulated controls (**Figure 3G**), indicating that the reduction in mitochondrial mass was proportionally greater than the decrease in mitochondrial membrane potential. Corresponding fold-change values relative to unstimulated controls for all metabolic parameters are shown in **Supplementary Figure 3**, confirming the consistency of these changes across donors.

To determine whether this altered mitochondrial profile was associated with increased mitochondrial oxidative stress, we measured mitochondrial ROS production, finding no significant differences between PgOMV-stimulated and unstimulated neutrophils (**Supplementary Figure 4A**). Neutrophil viability was assessed by Annexin V/PI staining after 3 h of stimulation, corresponding to the time point used for most metabolic analyses. Untreated neutrophils exhibited the expected spontaneous apoptosis under culture conditions, whereas PgOMV stimulation did not increase cell death and showed a tendency to reduce spontaneous apoptosis, consistent with previous reports demonstrating that neutrophil activation delays constitutive apoptosis. These findings indicate that the reductions in mitochondrial mass and membrane potential are not secondary to decreased cell viability (**Supplementary Figure 4B**).

### PgOMV pretreatment of neutrophils enhances *P. gingivalis* internalization and persistence

**Figure 4:**
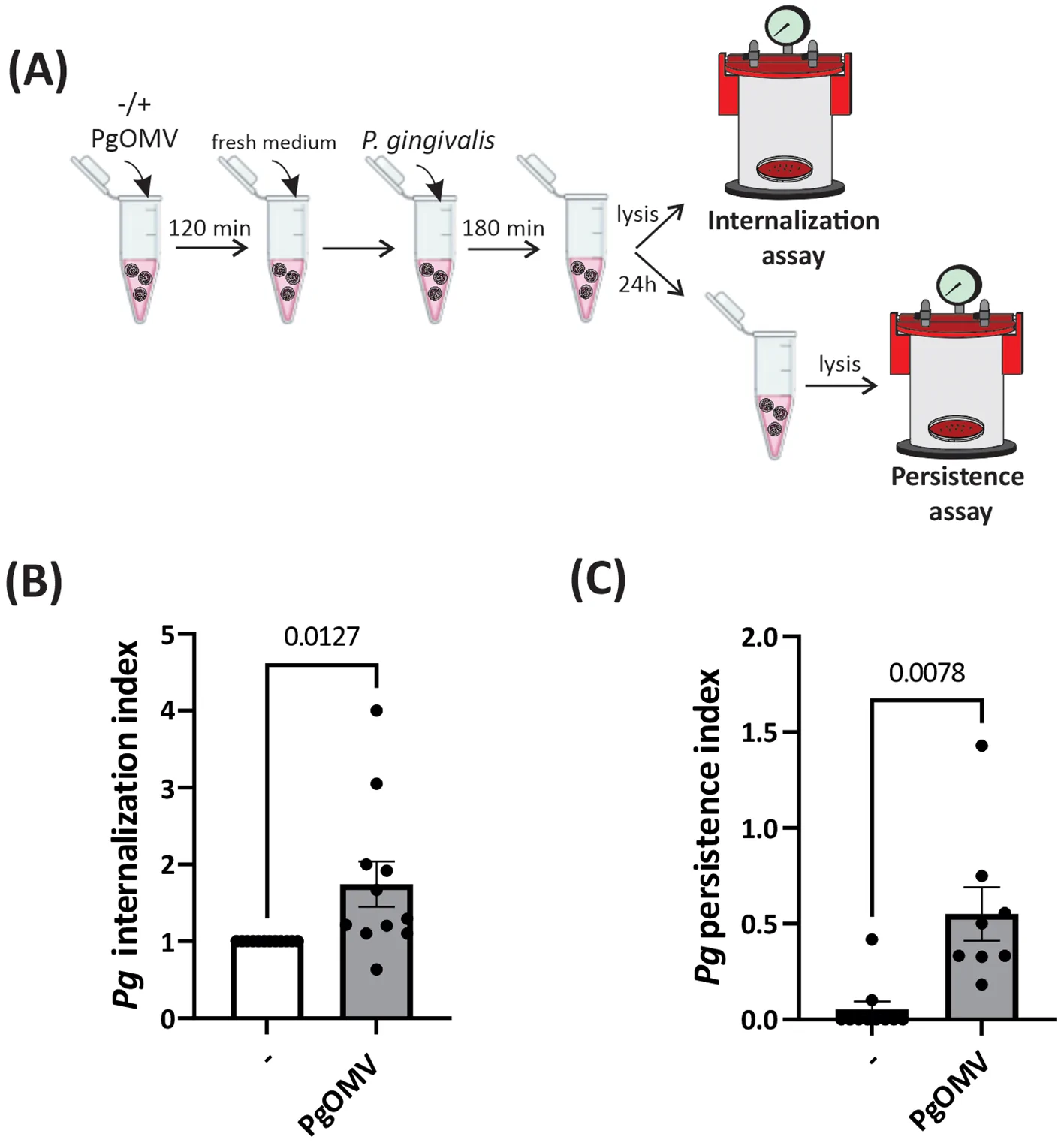
PgOMV pretreatment induces *P. gingivalis* internalization and persistence in neutrophils. **(A)** *P. gingivalis* internalization index and persistence index in neutrophils pre-treated for 120 min with PgOMV. Untreated neutrophils **(-)** were used as controls. **(B)** The internalization index was evaluated as the number of internalized bacteria, determined by counting CFUs recovered on agar plates after the internalization assay, n=8. **(C)** The persistence index was calculated as the ratio between the number of intracellular bacteria recovered after 24 h and the number of internalized bacteria measured in **(B)**, n=6. Both assays were performed in parallel. Results are expressed as mean ± SEM. Each point represents an independent experiment, with two technical replicates per experiment selected based on countable, non-overlapping colonies and consistent serial dilutions. A Wilcoxon test was used.

To evaluate whether the neutrophil state induced by PgOMV exposure influences subsequent bacterial infection, neutrophils were pretreated with PgOMV for 120 min and then challenged with viable *P. gingivalis* bacteria to assess bacterial internalization and persistence (**Figure 4A**). Neutrophils pretreated with PgOMV showed a significantly higher internalization and persistence index compared to untreated controls (**Figures 4B, C**).

### Conditioned media from PgOMV treated neutrophils impair trophoblast migration

**Figure 5:**
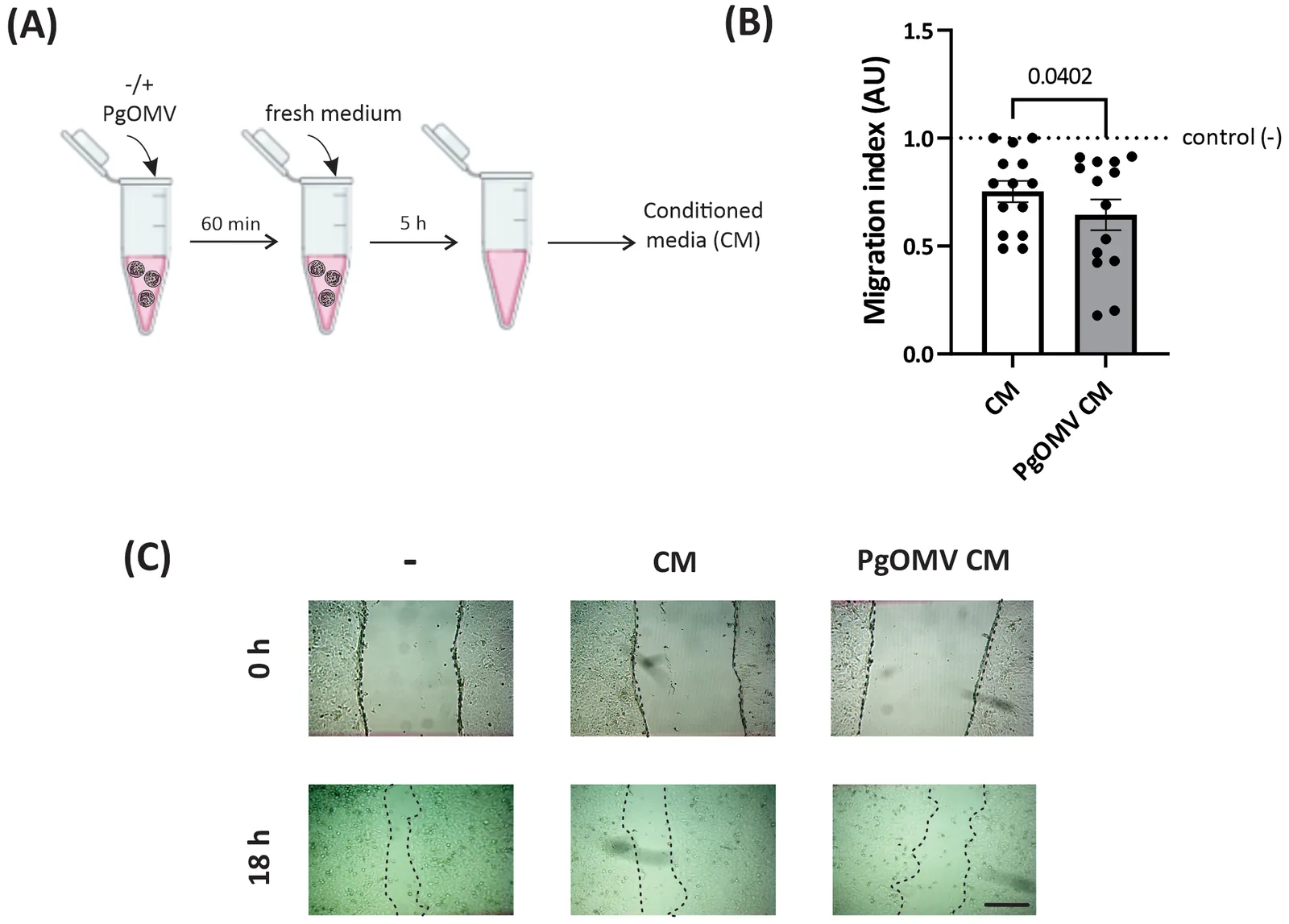
Neutrophils exposed to PgOMV diminish trophoblast cell migration. **(A)** Neutrophils were stimulated in absence or presence of 5 μg/mL PgOMV during 60 min, then cells were washed twice with PBS to remove PgOMV in the medium. Cells were incubated during 5 h more and conditioned media (CM) from neutrophils treated with PgOMV (PgOMV CM) or not (CM) were collected and stored at -20 °C until use. **(B)** HTR-8 trophoblast cells were grown until they reached confluence were then wounded and incubated for 0−18 h in RPMI 1640 medium supplemented with 2% FBS in absence (control) or presence of neutrophil CM or PgOMV CM. Microphotographs were taken at 0 and 18 h postscratching and closure was assessed. Values given are expressed as mean ± SEM. Neutrophils from each donor were stimulated with two independent PgOMV preparations obtained from different purification batches; therefore, two data points are shown for each donor. n = 7. A Wilcoxon test was used. **(C)** Microphotographs of a representative experiment are shown. Scale bar: 500 μm.

At the maternal-fetal interface, neutrophils contribute to tissue remodeling and vascular adaptation, processes that require tightly controlled inflammatory activation to preserve adequate trophoblast invasion and placental development (19). To determine whether the neutrophil phenotype induced by PgOMV could indirectly alter trophoblast behavior, neutrophils were stimulated for 60 min with PgOMV, then washed twice and incubated for 5 h more and conditioned media (CM) was collected and used in a wound healing assay with HTR-8/SVneo trophoblast cells (**Figure 5A**). Conditioned media from PgOMV treated neutrophils further impaired trophoblast migration compared to conditioned media from unstimulated neutrophils (-), supporting the idea that OMV driven neutrophil reprogramming may indirectly contribute to placental dysfunction. (**Figure 5B**), as illustrated by representative microphotographs of the wound healing assay (**Figure 5C**).

### Oxidative stress reshapes PgOMV immunomodulatory activity enhancing intracellular bacterial persistence in neutrophils

**Figure 6:**
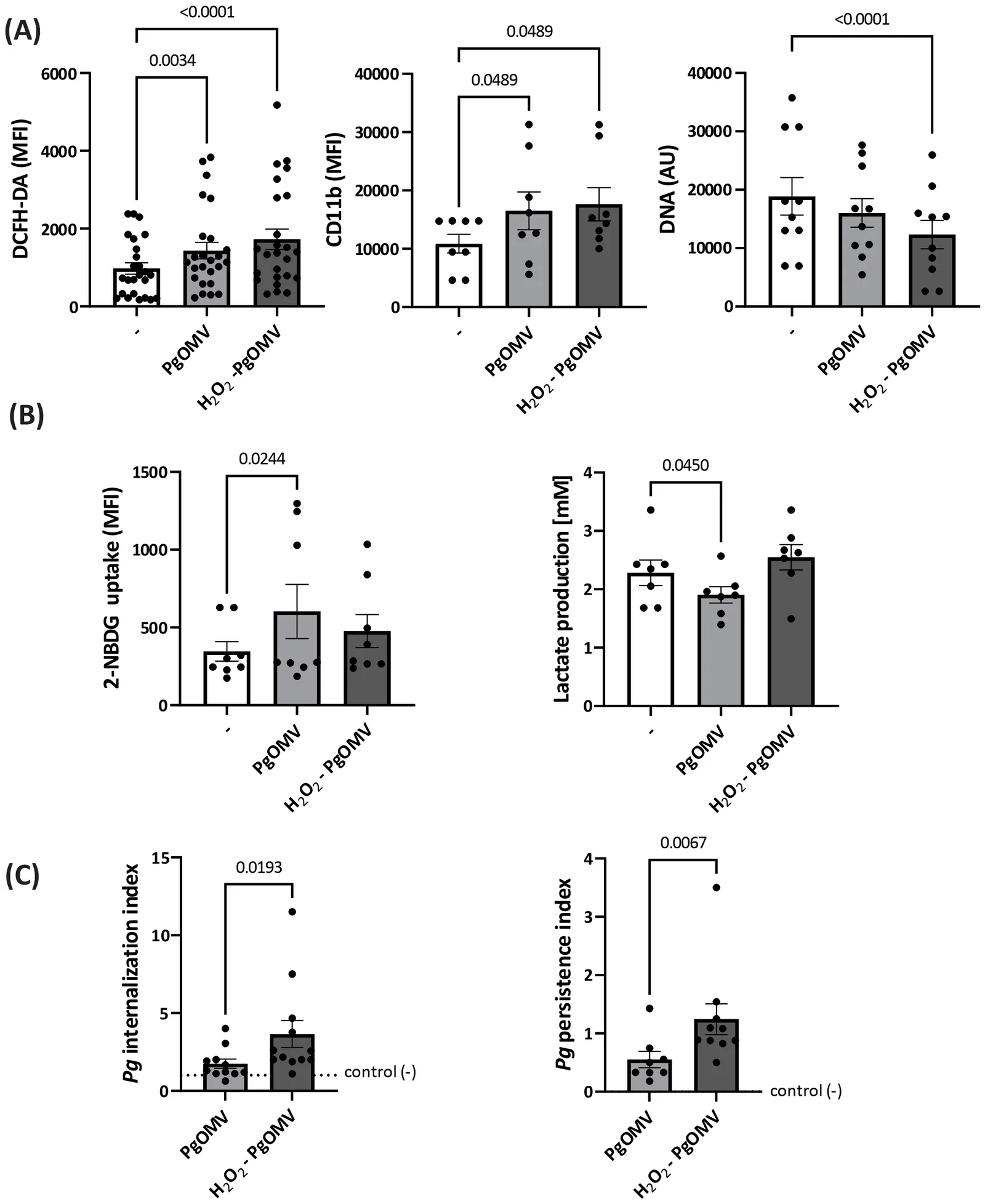
H_2_O_2_-PgOMV induce a more permissive neutrophil phenotype for *P. gingivalis* internalization and survival. Neutrophils were stimulated with 5 μg/mL PgOMV or H_2_O_2_-PgOMV as mentioned in Material and Methods. **(A)** ROS production, CD11b expression and DNA release were evaluated as before. **(B)** Glucose uptake was determined in neutrophils after 60 min of culture by flow cytometry using 2-NBDG, whereas lactate release was measured in supernatant after 6 h of stimulus. For CD11b, DNA release, and glucose uptake, neutrophils from each donor were stimulated with two independent PgOMV and H_2_O_2_-PgOMV preparations obtained from different purification batches; therefore, two data points are shown for each donor. Biological replicates were n = 4 for CD11b, n = 5 for DNA release, n = 4 for glucose uptake, and n = 12 for ROS production. **(C)** Neutrophils pre-treated for 120 min with PgOMV or H_2_O_2_-PgOMV were infected with *P.gingivalis* and the internalization index and persistence index was studied as before. A Friedman test with Dunn’s correction was used in A and B, whereas a Wilcoxon matched-pairs signed-rank test was used in C.

Finally, we investigated whether oxidative stress conditions, previously shown to modify PgOMV physicochemical properties and protein cargo (7), also altered the immunomodulatory activity on neutrophils. We therefore compared the effects of control PgOMV and H_2_O_2_-PgOMV. To address this, we compared neutrophil responses to OMVs derived from bacteria cultured under basal conditions (PgOMV) or exposed to oxidative stress (H_2_O_2_-PgOMV). Both OMV preparations induced comparable increases in ROS production and CD11b surface expression, and neither promoted extracellular DNA release, indicating that oxidative stress-dependent remodeling did not substantially alter the capacity of PgOMV to induce neutrophil activation (**Figure 6A**). In contrast, differences emerged in metabolic-associated parameters. While basal PgOMV increased glucose uptake and reduced lactate release, these effects were not observed in neutrophils stimulated with H_2_O_2_-PgOMV, where glucose uptake and lactate secretion remained comparable to unstimulated controls (**Figure 6B**). Although classically activated neutrophils typically enhance glycolysis with a concomitant rise in lactate production, our results indicate a distinct profile, potentially reflecting a redirection of glucose-derived metabolites toward biosynthetic or redox-related pathways rather than canonical glycolytic activation, a pattern that has been described in neutrophil subsets adapting to specific microenvironmental or infectious cues.

These differences were accompanied by altered responses to bacterial challenge. Neutrophils pretreated with H_2_O_2_-PgOMV displayed significantly increased intracellular internalization and persistence of *P. gingivalis* compared to cells pretreated with basal PgOMV (**Figure 6C**), indicating that oxidative stress-derived vesicles promote a more permissive neutrophil phenotype for bacterial survival.

## Discussion

In this study, we demonstrate that *P. gingivalis* OMV actively reprogram human neutrophils through a complex process involving oxidative activation, metabolic changes, impaired extracellular antimicrobial responses, and altered communication with trophoblast cells. Importantly, we further show that oxidative stress experienced by the bacteria reshapes the immunomodulatory properties of released OMV, modifying neutrophil bacterial internalization and persistence. Together, these findings support the concept that PgOMV are not passive byproducts of bacterial growth but dynamic effectors capable of fine-tuning host innate immune responses according to environmental conditions.

PgOMV triggered neutrophil activation, evidenced by increased CD11b expression and ROS production, yet did so without engaging CD14, a pattern that contrasts with OMV effects previously documented in macrophages (20). PgOMV reduces CD14 expression and results in macrophage hyporesponsiveness to bacterial challenge. The absence of this effect in neutrophils suggests that these cells sense PgOMV through alternative pattern recognition pathways, most likely TLR2 and complement receptors, which are the predominant OMV-sensing mechanisms in neutrophils. This cell-type specificity underscores that OMV-host interactions are not uniform across innate immune cells and that the functional outcome of vesicle recognition depends critically on the receptor repertoire of the target cell. Beyond CD14, other surface and functional markers linked to dysfunctional or immunosuppressive neutrophil subsets, including PD-L1, ARG1, LOX-1, CXCR4, and altered CD62L or CD16 expression, could offer a more comprehensive picture of the permissive phenotype described here and represent promising candidates for future characterization of the neutrophil state induced by PgOMV.

Among the most striking findings of this study is the failure of PgOMV to induce NET formation, combined with their capacity to actively degrade pre-existing NETs through DNase activity. This dual strategy, suppressing new NET formation while dismantling existing ones, represents a potentially powerful mechanism of immune evasion. DNase activity has been reported for *P. gingivalis* whole bacteria (21), and our results extend this to OMVs as vehicles for extracellular delivery of this virulence factor, enabling the bacterium to act at a distance from the site of infection. These observations are also consistent with previous findings by Du Teil Espina et al. (22), who demonstrated that PgOMV coat and activate human neutrophils, promoting degranulation without inducing neutrophil death. They also found that antibacterial granule-derived components LL-37 and myeloperoxidase are subsequently degraded by OMV-associated gingipains, thereby preserving bacterial survival. Together, these findings support a model in which PgOMV induce a partially activated yet functionally permissive neutrophil phenotype, in which inflammatory activation is maintained while key antimicrobial effector mechanisms are neutralized. The functional significance of these profiles is important in the context of periodontitis, where neutrophil infiltration is a defining feature of disease; by disarming NET-mediated killing, PgOMV may facilitate bacterial persistence within inflamed periodontal tissues and at distal sites including the maternal-fetal interface.

The metabolic-associated changes induced by PgOMV adds another dimension to this evasion strategy. Increased glucose uptake alongside reduced lactate secretion suggests that glucose utilization may be differentially regulated under these conditions. Although classically activated neutrophils are generally considered highly glycolytic cells, accumulating evidence indicates that neutrophils display considerable metabolic plasticity and can adopt distinct metabolic programs depending on the activating stimulus and tissue microenvironment (14, 24). In particular, activated neutrophils may redirect glucose-derived carbons toward the oxidative pentose phosphate pathway to sustain NADPH generation and ROS production rather than maximizing lactate production through glycolysis (23). Such a mechanism is consistent with our observation of increased ROS despite reduced extracellular lactate, although this possibility remains speculative because metabolic fluxes were not directly assessed. Importantly, this program appears to sustain activation-associated responses (ROS, CD11b) without supporting the full antimicrobial arsenal. Taken together with the absence of NET induction and the DNase activity associated with PgOMV, these observations support a neutrophil state characterized by inflammatory activation accompanied by impaired extracellular antimicrobial responses, consistent with emerging evidence indicating that metabolic programs contribute to shaping neutrophil effector functions (24). Although our findings support the existence of metabolic adaptations associated with PgOMV stimulation, our present study did not directly evaluate metabolic fluxes or specific pathway activity. Therefore, the proposed redistribution of glucose metabolism toward pathways linked to redox balance and NADPH generation should be interpreted as a functional association rather than definitive metabolic pathway characterization. Future studies combining metabolic flux analysis and targeted metabolomics will help further define the metabolic adaptations induced by PgOMV in neutrophils.

PgOMV stimulation also promoted lipid droplet accumulation and reduced mitochondrial mass together with alterations in mitochondrial membrane potential. Lipid droplets are increasingly recognized as dynamic inflammatory organelles that participate in redox regulation, arachidonic acid metabolism, and the generation of lipid mediators (25). Their accumulation in OMV-stimulated neutrophils may therefore represent a metabolic adaptation associated with inflammatory activation. Notably, lipid droplet-derived mediators are secreted by activated neutrophils (26) and could represent candidate components of the conditioned media that impaired trophoblast migration in our model. The observation that both mitochondrial mass and membrane potential were reduced after PgOMV stimulation, yet the membrane potential-to-mass ratio was increased, indicates that mitochondrial content decreased proportionally more than membrane potential. Importantly, mitochondrial ROS production was not increased under these conditions, suggesting that mitochondria are unlikely to be the major source of the increased intracellular ROS observed after PgOMV stimulation. Instead, these findings are consistent with a remodeling of the mitochondrial compartment rather than overt mitochondrial dysfunction. Although this parameter alone does not explain the permissive neutrophil phenotype, it forms part of a broader metabolic adaptation that accompanies neutrophil activation while extracellular antimicrobial functions remain impaired. Whether these mitochondrial changes contribute directly to bacterial persistence or represent a parallel adaptation warrants further investigation.

Neutrophils preconditioned with PgOMV showed significantly increased *P. gingivalis* internalization and persistence, indicating that prior PgOMV exposure is associated with a more permissive intracellular environment for both bacterial entry and survival. Consistent with this, engagement of DC-SIGN by *P. gingivalis* Mfa-1 fimbriae has been shown to promote evasion of antibacterial autophagy and lysosome fusion in myeloid dendritic cells, enabling intracellular persistence within single-membrane vesicles (27). Whether analogous mechanisms operate in PgOMV preconditioned neutrophils remains to be determined.

Conditioned media from PgOMV-stimulated neutrophils further impaired trophoblast migration suggests that PgOMV exposure is associated with changes in the neutrophil secretory profile with functional consequences at the maternal-fetal interface. Potential mediators may include inflammatory lipid mediators associated with lipid droplet accumulation, as well as cytokines, proteases, or oxidative factors released following PgOMV stimulation. Although we cannot completely rule out that a minimal fraction of PgOMV remained associated with the neutrophil surface despite extensive washing, we consider it unlikely that such a small residual amount would account for the impairment in trophoblast migration observed with PgOMV-conditioned media; nonetheless, a direct contribution of residual PgOMV cannot be formally excluded. Defective trophoblast migration and impaired spiral artery remodeling are recognized features of pregnancy complications such as preeclampsia and placental insufficiency (28), conditions in which dysregulated neutrophil activation has also been implicated (29, 30). In this context, our findings support the idea that *P. gingivalis* OMV may contribute to placental dysfunction not only through direct interactions with trophoblast cells as we previously described (6), but also indirectly through neutrophil-mediated inflammatory mechanisms.

The comparison between basal PgOMV and H_2_O_2_-PgOMV revealed that both preparations induced comparable neutrophil activation responses yet differed in their effects on metabolic parameters and bacterial persistence. In a previous work, we demonstrate differences in the proteome of PgOMV produced after an exposure to H_2_O_2_ compared with those from control cultures (7). Importantly, several proteins with fimbrial and adhesin domains and a Von Willebrand factor type A domain protein were found only in H_2_O_2_-PgOMV in line with the increase of invasion found in this work. Moreover, a protein homologous to an internalin in *Listeria monocytogenes*, that mediates the cell invasion through the interaction with E-cadherin and tyrosine kinase receptor (MET), was also found only in the surface of PgOMV derived from Pg treated cultures, indicating that the cargo of stressed bacteria impact even more in the host cells (31, 32). This differential cargo provides a plausible compositional basis for the distinct functional outcomes observed between basal and H_2_O_2_-PgOMV in neutrophils, comparable activation but divergent metabolic responses and enhanced bacterial internalization and persistence with H_2_O_2_-PgOMV, linking the oxidative stress-induced remodeling of vesicle composition to its enhanced capacity to promote a permissive neutrophil phenotype.

Neutrophils preconditioned with PgOMV showed significantly increased *P. gingivalis* internalization and persistence, establishing that the altered neutrophil state created by OMV exposure is functionally permissive for bacterial entry. Notably, this effect is cell-type specific and context-dependent. In trophoblast cells, H_2_O_2_-PgOMV lost the pro-invasive activity of basal OMV (7). In neutrophils, by contrast, H_2_O_2_-PgOMV induces bacterial internalization while extending persistence. This divergence likely reflects fundamental differences in the intracellular biology of trophoblasts versus neutrophils that may include their respective oxidative capacities, phagolysosomal activity, and metabolic flexibility. The finding underscores that the functional consequences of stress-dependent OMV remodeling cannot be extrapolated across cell types and must be evaluated in the specific cellular context relevant to each infection site.

In summary, our study shows that PgOMV are associated with a neutrophil phenotype characterized by inflammatory activation alongside impaired antimicrobial responses, changes in metabolic-associated parameters, and increased permissiveness to intracellular bacterial survival. Oxidative stress further modifies these properties in a cell-type-specific manner. These findings contribute to growing evidence linking periodontal pathogens and their derived vesicles to dysregulation of innate immune responses relevant to both local and systemic disease contexts, including adverse pregnancy outcomes. As the present findings derive entirely from an *in vitro* model, the proposed link between PgOMV-driven neutrophil reprogramming and adverse pregnancy outcomes should be regarded as a plausible mechanistic hypothesis rather than an established causal pathway, and its physiological relevance remains to be validated *in vivo*. Future studies should address the specific OMV cargo components responsible for each functional effect and evaluate whether these mechanisms operate *in vivo* in relevant infection models.

## Data Availability

The raw data supporting the conclusions of this article will be made available by the authors, without undue reservation.
