# Supplementary material for: Porphyromonas gingivalis outer membrane vesicles induce an activated but permissive neutrophil phenotype
Source: Front Immunol. 2026 Sep 3;17:1896511. doi: 10.3389/fimmu.2026.1896511 (PMC13581572; doi:10.3389/fimmu.2026.1896511)
Supplement: Supplementary file 1 [file SupplementaryFile1.docx]

***Porphyromonas gingivalis* outer membrane vesicles induce an activated but permissive neutrophil phenotype**

Ailén Fretes^1^*, Brenda Lara^1^*, Guillermina Calo^1^, Ignacio Rojas Campión^1^, Lourdes^1^ Materazzi, Florencia Sabbione^2^, Stefania A. Robaldi^1^, Maria Constanza Pautasso^1^, Claudia Pérez Leirós^1^, Daiana Vota^1^, Paula M. Tribelli ^1#^, Vanesa Hauk ^1#^

**Supplementary figure S1**

**Supplementary Figure S1. Representative gating strategy for flow cytometry analyses.** a) Neutrophils were first identified based on their characteristic forward scatter (FSC) and side scatter (SSC) properties to exclude debris. Singlets were then selected using FSC-A versus FSC-W to eliminate doublets and cell aggregates. b) Neutrophil purity was assessed separately by CD14 staining to determine monocyte contamination. Since CD14 expression on monocytes is more than one order of magnitude higher than on neutrophils, this approach enables the identification and quantification of monocyte contamination within the isolated neutrophil population. Cell preparations containing more than 2% CD14⁺ cells were excluded from the study, resulting in a neutrophil purity of >98% in all experiments. In the representative plot, monocyte contamination was 0.37%, confirming the purity of the sample as acceptable for downstream experiments.

**Supplementary figure S2**

**Supplementary Figure S2. Positive control for NET formation.**

Neutrophils were stimulated with 50 nM PMA for 210 min and (A) elastase activity and (B) extracellular DNA release were measured in supernatants as described for Figure 2, confirming that the assay reliably detects NET formation under our experimental conditions. Values are mean ± SEM.

**Supplementary figure S3**

**Supplementary Figure S3. Fold-change representation of metabolic parameters shown in Figure 3.**

Data from Figure 3 are expressed as fold change relative to unstimulated controls (set to 1) to better illustrate the magnitude of PgOMV-induced changes. (A) 2-NBDG uptake. (B) Lactate production. (C) Bodipy 493/503 (lipid droplets). (D) Bodipy-FL C12 uptake (long-chain fatty acid uptake). (E) Mitospy green (mitochondrial mass). (F) Mitotracker CMXRos (mitochondrial membrane potential). Values are mean ± SEM. Wilcoxon matched-pairs signed-rank test was used.

**Supplementary figure 4**

 **Supplementary Figure S4. Mitochondrial ROS production and neutrophil viability after PgOMV stimulation.**

(A) Mitochondrial ROS was measured by MitoSOX red staining in neutrophils stimulated with 5 μg/mL PgOMV, expressed as raw MFI and as fold change relative to unstimulated controls, with a representative histogram shown at right. No significant differences were observed between conditions. (B) Representative flow cytometry plots of Annexin V/Propidium Iodide staining in unstimulated (-) and PgOMV-stimulated neutrophils for 3h, used to assess cell viability.
